# Identification of Sequence Variants in Genetic Disease-Causing Genes Using Targeted Next-Generation Sequencing

**DOI:** 10.1371/journal.pone.0029500

**Published:** 2011-12-21

**Authors:** Xiaoming Wei, Xiangchun Ju, Xin Yi, Qian Zhu, Ning Qu, Tengfei Liu, Yang Chen, Hui Jiang, Guanghui Yang, Ruan Zhen, Zhangzhang Lan, Ming Qi, Jinming Wang, Yi Yang, Yuxing Chu, Xiaoyan Li, Yanfang Guang, Jian Huang

**Affiliations:** 1 Beijing Genomics Institute at Shenzhen, Shenzhen, China; 2 Shanghai-Ministry Key Laboratory of Disease and Health Genomics, National Engineering Center for Biochip at Shanghai, Shanghai, China; Auburn University, United States of America

## Abstract

**Background:**

Identification of gene variants plays an important role in research on and diagnosis of genetic diseases. A combination of enrichment of targeted genes and next-generation sequencing (targeted DNA-HiSeq) results in both high efficiency and low cost for targeted sequencing of genes of interest.

**Methodology/Principal Findings:**

To identify mutations associated with genetic diseases, we designed an array-based gene chip to capture all of the exons of 193 genes involved in 103 genetic diseases. To evaluate this technology, we selected 7 samples from seven patients with six different genetic diseases resulting from six disease-causing genes and 100 samples from normal human adults as controls. The data obtained showed that on average, 99.14% of 3,382 exons with more than 30-fold coverage were successfully detected using Targeted DNA-HiSeq technology, and we found six known variants in four disease-causing genes and two novel mutations in two other disease-causing genes (the STS gene for XLI and the FBN1 gene for MFS) as well as one exon deletion mutation in the DMD gene. These results were confirmed in their entirety using either the Sanger sequencing method or real-time PCR.

**Conclusions/Significance:**

Targeted DNA-HiSeq combines next-generation sequencing with the capture of sequences from a relevant subset of high-interest genes. This method was tested by capturing sequences from a DNA library through hybridization to oligonucleotide probes specific for genetic disorder-related genes and was found to show high selectivity, improve the detection of mutations, enabling the discovery of novel variants, and provide additional indel data. Thus, targeted DNA-HiSeq can be used to analyze the gene variant profiles of monogenic diseases with high sensitivity, fidelity, throughput and speed.

## Introduction

Genetic disorders are diseases caused by abnormalities in an individual's genome, including single-nucleotide variants (SNVs), small inversion and deletion (indel) and gross chromosomal abnormalities. Genetic disorders can be divided into single-gene disorders and multifactorial and polygenic disorders. Since the human genome project was completed in 2003, many other projects have been launched to identify genomic variants [1], including the 1000 Human Genomes Project (http://www.1000genomes.org/), the International HapMap Project [2], and the Cancer Genome Anatomy Project (http://cgap.nci.nih.gov/). To date, more than 100,000 mutations have been stored in the Human Gene Mutation Database (HGMD) at the Institute of Medical Genetics in Cardiff; 55.8% of these mutations are missense or nonsense mutations, and the others are indel and splicing mutations. More than 8,000 mutations have been added to the HGMD per year for the last 6 years. According to statistics reported by the OMIM database, there are over 6,000 single-gene disorders related to 12,000 genes with different variants. Extensive re-sequencing is required to explore the sequences of many disease-associated genes at the sequence and structural level.

The Sanger DNA sequencing method, which is well known for both the read length and accuracy of sequencing it provides, has been considered to represent the gold standard for screening variants of genes of interest in many scientific fields for both research and medical applications. When multiple genes need to be analyzed for mutations simultaneously, the gathering of information becomes laborious, expensive and time consuming using the Sanger method. Hence, this technique is not suitable for the identification of a large number of sequence variants in massive samples in one experiment. Since 2005, several next-generation sequencing (NGS) platforms have been established, including the Illumina HiSeq2000, the Roche 454, and the ABI SOLiD platforms, all of which are able to generate a massive amount of sequence data [3]. Although NGS technology can be used to analyze gene variants with high throughput screening, its high cost still prevents its application in whole human genome resequencing projects. Therefore, targeted sequence analysis of interesting genomic regions is an attractive approach that can be used to develop novel applications. The combination of targeted enrichment systems with NGS has been used in research on both complex diseases and single-gene disorders [4]–[6]. Several sophisticated target-capture methods have been developed to either capture the entire human exome or provide customized capture of regions of interest [7]–[10], including the Agilent SureSelect target-enrichment system and the Nimblegen solid-phase hybrid array.

NGS platforms, coupled with DNA target-capture using solid-phase arrays and multiple sample barcoding technology, have become a powerful tool for identifying genetic variants. Recent explorations into hundreds of candidate disease genes in different individuals have demonstrated the possibility of low-cost genetic screening [11], [12]. In this study, we have designed a custom high-density, solid-phase array (Roche NimbleGen, Inc., Madison, WI, USA) to capture 193 distinct genes involved in 103 different genetic diseases. After enrichment, high-throughput sequencing using the Illumina HiSeq2000 Analyzer (Illumina, San Diego, CA, USA) was performed to analyze the variants of disease-causing genes in different patient samples in one sequencing lane using the sample barcoding method. Next, we used the Sanger DNA sequencing method followed by PCR amplification and MOLDI-TOF-MS to confirm the results obtained from NGS. Our current findings suggest that this targeted DNA-HiSeq technology can be used to detect variants of disease-causing genes with high fidelity, high throughput, high speed and low cost.

## Methods

### Patients and controls

A total of 107 blood samples were used in this study from 7 clinical cases: one with hereditary spastic paraplegia with thin corpus callosum (HSP-TCC, MIM# 604360), one with X-linked ichthyosis (XLI, MIM# 308100), one with Marfan syndrome (MFS, MIM# 154700), one with Wilson disease (MIM# 277900), one with Duchenne muscular dystrophy (DMD, MIM# 310200) and two patients with β-thalassemias (MIM# 603902) (Table 1), and 100 normal human adults as controls. In addition, 3 samples derived from YH DNA (C1), one additional normal human adult (C2) and the mother (C3) of the DMD proband (P7) were analyzed (Table 1). Written informed consent was obtained from all of the participants, who each provided 5 ml of blood for the study. This project and the protocols for the investigation involving human tissues were approved by the ethics committee of the Beijing Genomics Institute at Shenzhen.

**Table 1:** Overview of performance of Targeted DNA-HiSeq.

Sample	Gender	Total reads	mapped reads	Capture efficiency	Coverage of target region	Mean coverage
C1	Male	10 481 008	7 496 661	71.53%	99.57%	393.97
C2	Female	15 185 260	11 080 462	72.97%	99.69%	570.11
C3	Female	14 058 272	10 040 422	71.42%	99.58%	515.10
P1	Male	12 485 604	8 843 811	70.83%	99.35%	455.99
P2	Male	11 134 408	7 916 144	71.10%	99.52%	407.10
P3	Female	6 932 866	5 124 501	73.92%	99.55%	264.84
P4	Female	11 173 742	7 895 879	70.66%	99.62%	404.24
P5	Male	5 981 308	4 403 481	73.62%	99.48%	226.57
P6	Female	9 444 410	7 044 896	74.59%	99.52%	362.23
P7	Male	13 887 662	9 836 275	70.83%	99.51%	505.21

### Capture array design, library construction and next-generation sequencing

A custom capture array (NimbleGen, Roche) was designed to capture all exons (3382), splice sites and the immediately adjacent intron sequences of 193 genes known to be associated with three main types of hereditary diseases according to GeneReviews (NCBI), including single-gene disorders, malignant arrhythmias and cardiomyopathies, as well as familial malignant neoplasms.

The methods used for DNA target capture, enrichment and elution followed previously described protocols with minor modifications (Roche NimbleGen, Inc.). Briefly, genomic DNA from peripheral blood was extracted from the 110 samples using the QIAamp DNA BloodMiNi Kit (Qiagen, Hilden, Germany), and fragmentation of the DNA into fragments ranging from 200 bp to 300 bp was performed using an ultrasonoscope (Covaris S2, Massachusetts, USA). Next, 1 µg of purified DNA (quantified by NanoDrop) was treated with T4 DNA polymerase, T4 phosphonucleotide kinase and the Klenow fragment of *Escherichia coli* DNA polymerase to fill 5′ overhangs and remove 3′ overhangs. Terminal A residues were added following a brief incubation with dATP and the Klenow 3′-5′ exo-enzyme following standard Illumina protocols [13]. Adapter oligonucleotides from Illumina (single reads) were ligated to the ends. After the ligation was completed, successful adapter ligation was confirmed by 4-cycle PCR using a high-fidelity polymerase with PCR primers containing a custom-synthesized barcode sequence (8 bp) as a sample index signature. PCR was used to generate a library for further analysis, and the DNA adapter-ligated and indexed fragments from 10 libraries were pooled and hybridized to the capture array for 72 h. After hybridization and washing, the DNA fragments bound to the array were eluted using 300 ml of elution buffer (Qiagen, Valencia, CA, USA) for each array. A gasket (Agilent) was applied and placed on a in-house constrcted thermal elution device for 20 min at 95°C. We repeated this process once, adding 200 ml of elution buffer (Qiagen). After hybridization of the sequencing primer, base incorporation was carried out on Illumina HiSeq2000 Analyzers (following the manufacturer's standard cluster generation and sequencing protocols) for 90 cycles of sequencing per read to generate paired-end reads including 90 bps at each end and 8 bps of the index tag. Image analysis and base calling were performed using the Illumina Pipeline.

### Data filtering and analysis pipeline

After the entire run was completed, image analyses, error estimation and base calling were performed using the Illumina Pipeline (version 1.3.4) to generate primary data. Indexed primers were used to identify the different reads from different samples in the primary data, and only reads that were perfectly matched to the theoretical adapter indexed sequences and reads that matched the theoretical primer indexed sequences with a maximum of three mismatches were considered to be acceptable reads. We then removed a few unqualified sequences from the primary data using a local dynamic programming algorithm, which included low-quality reads, defined as reads that contained more than 10 percent Ns in the read length, 50% reads with a quality value of less than 5 and with an average quality of less than 10 and adapter sequences including indexed sequence. The remaining sequences were termed as clean reads for further analysis.

The clean reads with a length of 90 bp were then subjected to alignment against the reference human genome from the NCBI database (Build 37) using the BWA (Burrows Wheeler Aligner) Multi-Vision software package [14]. SNPs and indels were identified using SOAPsnp software [15] and the GATK Indel Genotyper (http://www.broadinstitute.org/gsa/wiki/index.php/, The Genome Analysis Toolkit), respectively. Previously identified SNPs were determined using the NCBI dbSNP or HapMap databases. Known disease-causing mutations were identified from the Human Gene Mutation Database at the Institute of Medical Genetics in Cardiff (HGMD, http://www.ghmd.cf.ac.uk/) or from mutations previously reported in the literature.

### Sanger sequencing

To verify the DNA sequence variants (substitutions or indels) detected by NGS, we amplified the target sites and the flanking sequences of each patient's DNA template individually with specific primers designed using Primer6.0. We then sequenced the PCR products containing potential variants by the Sanger method to further ascertain the precision of the variants identified by NGS. Sequencing reactions were performed by mixing 5 µl of previously purified PCR products (ExoSAP-IT, USB, Cleveland, OH), 0.75 µM of primers and 1 µl of BigDye Terminator v1.1 Cycle Sequencing kit (Applied Biosystems, Foster City, CA) and were run in an ABI 3730 DNA Analyzer (Applied Biosystems).

### Real-time PCR

To further quantify the DNA copy number change for the DMD exon in specimens from DMD patients, the relative DNA copy number level for the DMD exon was measured by quantitative real-time PCR using the TaKaRa PCR Thermal Cycler Dice Detection System and SYBR green dye (TaKaRa, Japan) in three specimens (C2, C3 and P7) according to the protocol recommended by the manufacturer. A housekeeping gene, β-actin, was used as an endogenous control. Measurements were repeated at least three times to ensure the reproducibility of the results. The DNA copy number level for the DMD exon in each sample was compared with the level in control blood samples from normal adult females. The average Ct value of the β-actin gene was subtracted from the average Ct value for DMD for each sample: DMD ΔCt (average DMD Ct – average actin Ct), DMD ΔΔC (DMD ΔCt(P) - DMD ΔCt(N)). The fold change (2^-MDMΔΔCt^) in DMD copy number relative to β-actin copy number for each sample examined was calculated. A significant change was defined as a p value<0.05 [16]. In addition, we used SIFT software (http://sift.jcvi.org) to predict the pathogenicity of the single-nucleotide substitution variants.

## Results

### Sequence enrichment, high-throughput sequencing, mapping and coverage

**Figure 1:**
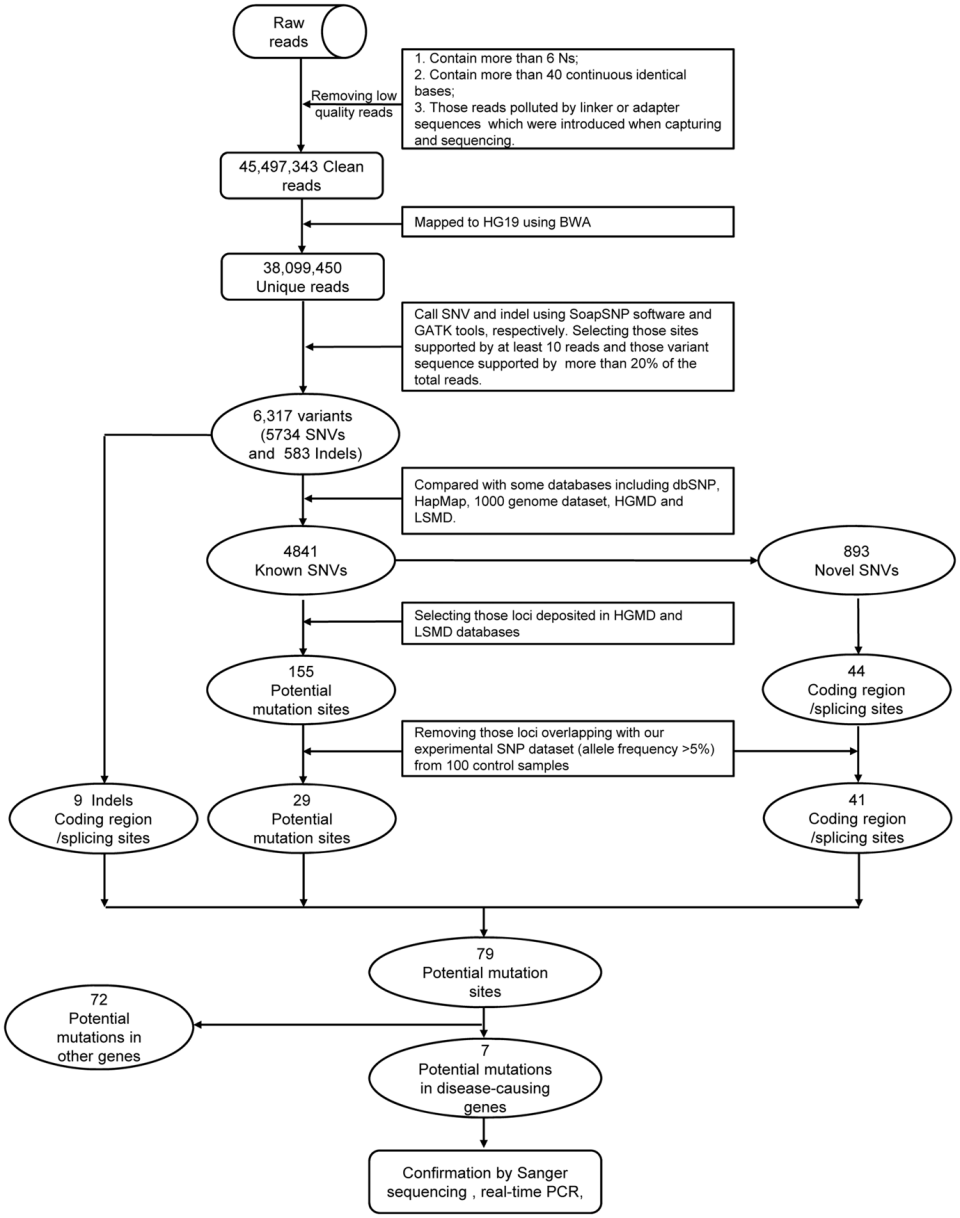
Flowchart for the process of screening and identifying variants in disease-causing genes by Targeted DNA-HiSeq.

**Figure 2:**
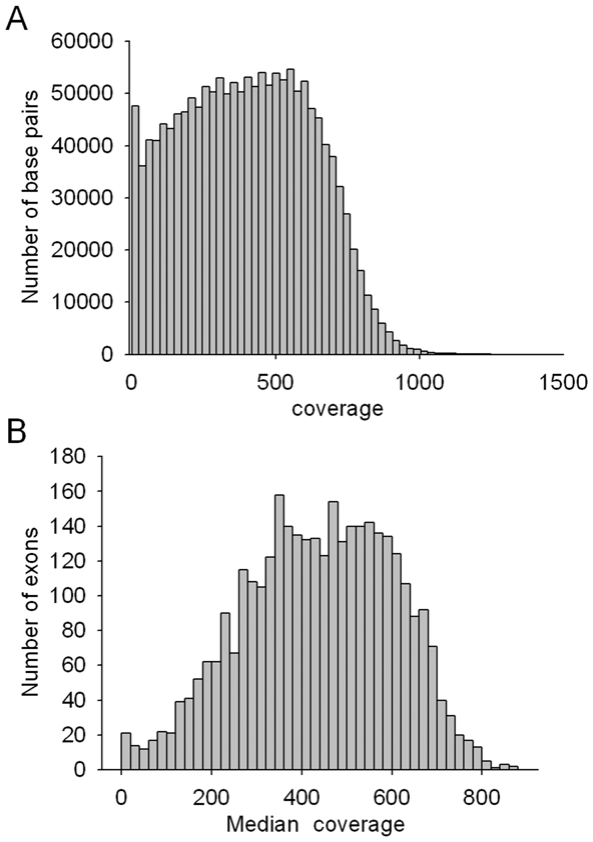
Distribution of sequence coverage. (A) The relationship between sequence coverage and each base pair of the sequenced exons in the 193 genes in YH DNA. (B) The relationship between median coverage and each exon number in all of the 10 samples sequenced in one lane.

To discover DNA variants of disease-causing genes involved in genetic disorders, we designed a unique high-density capture array to capture the 3382 exons and adjacent intron sequences of 193 genes causing distinct hereditary diseases (approximately 1.54 Mb, Table S1) that have a higher incidence in the Chinese population. Each DNA probe in the array is approximately 60 bp in length, and the probes can hybridize with all of the targeted exon sequences plus their flanking sequences (100 bp on each side of the exon, Table S2). The enriched DNA was sequenced at a single-base resolution using the Illumina HiSeq2000 Analyzer. To evaluate this technology, we collected seven samples from seven patients with six different genetic diseases resulting from six disease-causing genes as well as 100 samples from normal human adults as a control. Using Targeted DNA-HiSeq, we obtained a total of 5,981,308–15,185,260 high-quality reads encompassing 538,317,720–1,366,673,400 high-quality bases per patient (Table 1, Figure 1). After mapping to the reference human genome (HG19), 83.74% of the yielded clean reads could be uniquely matched to target regions, and 99.54% of the targeted region was covered in at least a 30-fold depth in each sample (Figure 2). The average coverage depth for exons was 410-fold, and the highest coverage depth for exons was 1000-fold (Table 1, Figure 2) across the seven samples. Thus, the coverage should have been adequate to reliably detect DNA variants within the majority of the targeted regions.

### Estimation of the accuracy of Targeted DNA-HiSeq

**Figure 3:**
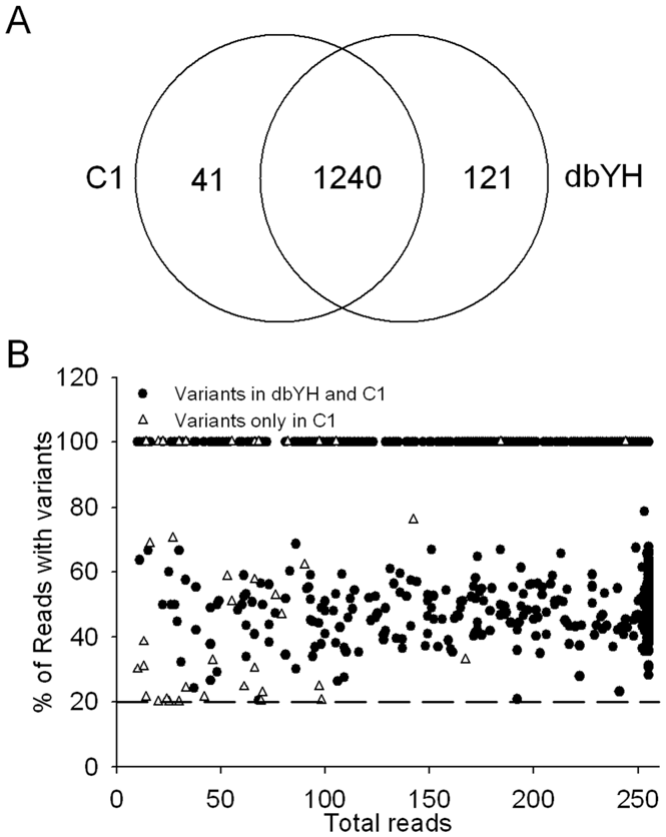
Estimation of the accuracy of Targeted DNA-HiSeq according to the following criteria: (1) sites are supported by at least 10 reads; (2) a variant sequence is supported by more than 20% of the total reads for a heterozygous genotype; and (3) a quality value of greater than 20 per base is obtained. (A) Venn diagrams of the number of variants in C1 as detected by Targeted DNA-HiSeq and in dbYH. Based on the reference genome, we found 1281 SNVs of 193 genes in YH DNA using Targeted DNA-HiSeq, of which 96.8% (1240/1281) were also identified in dbYH. (B) The distribution ratio of candidate variants in heterozygous genotypes between common variants (1240 variants) and specific variants (41 variants).

To estimate the accuracy of our method, we detected variants of 193 genes in YH DNA using our Targeted DNA-HiSeq technology and compared the results obtained with the results from whole-genome sequencing for the same 193 genes in the same DNA samples deposited in dbSNP (dbYH) [17]. The variants were considered to be candidate variants according to the following criteria: (1) the sites were supported by at least 10 reads; (2) the variant sequence was supported by more than 14% of the total reads; and (3) the quality value was greater than 20 per base [18]. Based on the reference genome, we found 1301 SNVs of 193 genes in YH DNA using Targeted DNA-HiSeq, of which 95.70% (1245/1301) were also identified in dbYH (Figure S1A). To further ascertain the appropriate ratio of candidate variants in heterozygous genotypes, we compared the distribution ratio of candidate variants in heterozygous genotypes between common variants (1245 variants) and specific variants (56 variants) (Figure S1A). The results showed that the ratio of candidate variants in heterozygous genotypes was more than 20% in 99.6% (1240/1245) of common variants (Figure S1B), suggesting that candidate variants should be supported by more than 20% of the total reads for heterozygous genotypes. Therefore, a new criterion was used to identify candidate variants. Based on these new standards, we found 1281 SNVs of 193 genes in YH DNA (C1) using Targeted DNA-HiSeq, of which 96.8% (1240/1281) were also identified in dbYH (Figure 3A and 3B). According to the current results, the predictive maximum false positive value (FPV) of Targeted DNA-HiSeq was 3.2% (41/1281). Finally, to further validate the accuracy of Targeted DNA-HiSeq, 52 candidate variants were randomly selected and were detected using the MALDI-TOF MS technique in YH DNA [19]. The results showed that all of the 52 variants identified by our method were confirmed by MALDI-TOF MS, suggesting that the Targeted DNA-HiSeq approach that was used in this study has high accuracy and can be applied to the analysis of clinical samples.

### Identification of sequence variants

To identify the variants of 6 disease-causing genes in 7 samples, the obtained high-quality reads were mapped to the reference sequence using BWA software, and the variants were then called using SOAPsnp software and GATK tools according to the above criteria. We finally identified 6317 variants, including 583 indels and 5734 substitutes. Among these variants, 84.4% (4841/5734) were termed as “known SNVs”, which were well known SNPs that had been deposited in the dbSNP database, HGMD, LSMD, and 1000 human genome dataset. To further test the candidate disease-related mutations, we selected the variants deposited in the HGMD or LSMD database, identifying 155 known disease-causing variants. In addition, we found 893 novel variants that had not been previously reported. Among the novel variants, we selected 44 variants that were predicted to be truncating (premature stop/frameshift/splice-site disruption) or to alter an amino acid, and the presence of two mutations in one gene, consistent with autosomal recessive inheritance (Figure 1). To find additional variants, GATK tools were employed to identify small insertions and small deletions. According to the criteria used to identify the point mutations described above, 9 candidate indels located in coding regions and splice sites were chosen for further analysis (Figure 1).

To exclude interference due to genetic polymorphism, we sequenced 193 genes in DNA from 100 blood samples from 100 human adults and constructed our own polymorphism database for these 193 genes. We then considered the variants that did not overlap with our annotated SNPs (with an allele frequency more than 5%) for the 193 genes as candidate novel mutations for further analysis, including 29 known substitutions, 41 novel substitutions and 9 novel indels. Out of the 79 total candidate mutations, 7 mutations occurred in 6 disease-causing genes (Figure 1 and Table 2). In addition, based on results from the OMIM database, there were an additional 72 candidate mutations in 49 genes unrelated to the six diseases (Table S3).

**Table 2:** Summary of mutations in seven disease-causing genes from seven genetic diseases.

Sample	Disease	Inheritance manner^a^	Gene	Exon	Nucleotide change	Amino acid change	SIFT^b^	Comment
P1	HSP-TCC	AR	SPG11	11	c.2163_2164insT	Frameshift defect	N.P	Known
P2	X-Linked ichthyosis	XR	STS	8	c.1099G>A	p.G367R	D	Novel
P3	Marfan syndrome	AD	FBN1	2	c.175T>C	p.C59R	D	Novel
P4	Wilson's disease	AR	ATP7B	8	c.2333G>T	p.R778L	N.P	Known
				12	c.2810detT	Frameshift defect	N.P	Known
P5	β-Thalassemia	AR	HBB	2	124_127delTTCT	Frameshift defect	N.P	Known
P6	β-Thalassemia	AR	HBB	−	IVS2+654C>T	Splicing defect	N.P.	Known
P7	DMD	XR	DMD	1	Del exon 1	Frameshift deletion	N.P.	Known

a Inheritance manner: AR (autosomal recessive), AD (autosomal dominant) and XR (X-linked recessive).

b SIFT classification: N.P (not predicted) and D (Damaging). Only the two novel variants were predicted with SIFT. SPG11 reference transcript NM_025137; STS reference transcript NM_000351; FBN1 reference transcript NM_000138; ATP7B reference transcript NM_000053; HBB reference transcript NM_000518; DMD reference transcript NM_000109.

**Figure 4:**
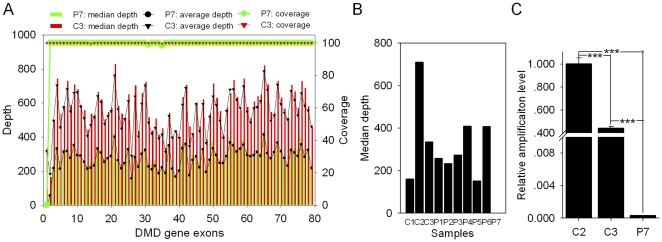
Detection of a deletion mutation of exon 1 in the DMD gene by targeted DNA-HiSeq. (A) Diagram of the median depth, average depth and sequencing coverage for all 79 exons in the DMD gene. The median/average depth and the sequencing coverage for the first exon of the DMD gene in patient P7 (proband) was close to 0 and was different from the coverage at exon 1 in the control, C3 (proband's mother). (B) The median depth for exon 1 of the DMD gene in all of the 10 samples sequenced in one lane. The median depth for exon 1 was close to 0 in P7, but not in the other patients. (C) Verification of the deletion mutation in P7 by real-time PCR (mean ± SEM, ANOVA analysis). The results showed that the relative amplification (RA) level of exon 1 is nearly 0, and the RA level in C3 is approximately half of the RA level in C2 (p<0.001). Genomic β-actin DNA was used as a loading control.

As is well known, a large deletions account for approximately 65% of abnormalities in individuals with DMD and 85% of those in patients with BMD (Becker muscular dystrophy, MIM# 300376) according to GeneReviews. Our results showed a lack of substitutions or indels in patient P7, who has DMD (Muscular Dystrophy, Duchenne Type). Therefore, to specifically address whether a large deletion was present in P7, we analyzed the depth of all exons of the DMD gene in the proband (P7) and his mother (C3) by Targeted DNA-HiSeq. Our results showed that the median read depth for the first exon of the DMD gene was 0 in P7, whereas the median depth for first exon of the DMD gene in the proband's mother (C3) was 334 fold (Figure 4A), and the average sequencing depth was more than 150 fold in the other 9 individuals sequenced in the same lane with P7 (Figure 4B). To address whether the low read depth at exon 1 in P7 was due to the capture or sequencing process, we analyzed the median depth of exon 1 of the DMD gene in the 100 control specimens, and no deletion was detected, suggesting that proband (P7) contains a deletion of exon 1 of the DMD gene that may be associated with DMD disease. To further confirm the deletion of exon 1 of the DMD gene in P7, we used quantitative PCR (qPCR) to detect the DNA copy number of the DMD gene in the proband (P7), his mother (C3) and a normal female control (C2), with the genomic β-actin gene being used as a loading control. Our results showed that the relative amplification level (RA level) was almost zero (0.032%) in P7, while the RA level was 444 (44.4%) in C2 when the value of C2 was normalized to 1000 (100%). There were significant differences between C2 and P7 as well as between C3 and P7 (p<0.001) (Figure 4C), suggesting that there was a homozygous deletion of exon 1 in the DMD gene in P7 and a heterozygous deletion in C3 (carrier status).

**Figure 5:**
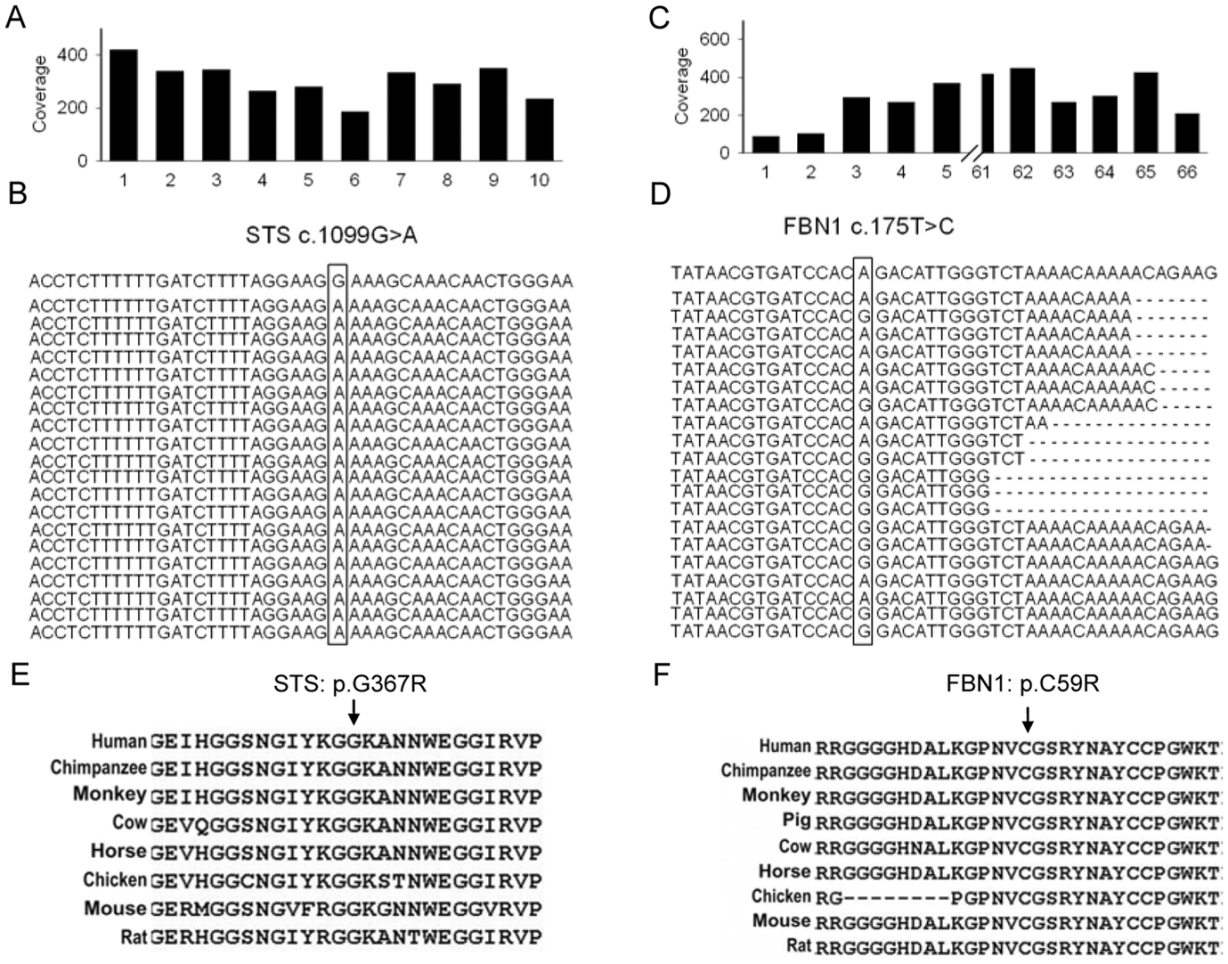
Identification of two novel mutations involved in X-linked ichthyosis and Marfan syndrome. (A) Median coverage for all 10 exons of the STS gene. (B) Representative reads aligned to the reference sequence (top) in exon 8 of the STS gene. The arrows indicate the locus with the substitution mutation (c.1099G>A), as supported by 248 high-quality reads. (C) Median coverage for all 66 exons of the FBN1 gene. (D) Representative reads aligned to the reference sequence (top) in exon 2 of the FBN1 gene. The arrows indicate the locus with the substitute mutation (c.175T>C), as supported by 243 high quality reads. (E) Evolutionary conservation analysis of the sequence with a variant (p. G367R) in the STS gene from 8 species by ClustalW alignment. (F) Evolutionary conservation analysis of the sequence with a variant (p.C59R) in the FBN1 gene from 8 species by ClustalW alignment.

In addition, among the 7 potential mutations in 6 disease-causing genes, two novel mutations were identified in two genes (STS and FBN1), which are involved in X-linked ichthyosis disease and Marfan syndrome, respectively (Table 2). Our sequencing results showed that all 10 of the exons of the STS gene (8.2 kb genomic intervals) exhibited an average coverage of 290 fold with a single base resolution (Figure 5A). A homozygous substitution mutation (c.1099G>A; p.G367R) was found in exon 8 of the STS gene in the DNA of a male patient (P2) that was supported by all 248 reads that spanned this site, and all of the 66 exons in the STS gene (∼25 kb genomic intervals) showed an average coverage of 300 fold with a single base resolution (Figure 5B). All of the 66 exons of the FBN1 gene (24.9-kb genomic intervals) presented an average coverage of 500 fold, with a single base resolution (Figure 5C). A substitution mutation (c.175T>C, p.C59R) was found in exon 3 of the FBN1 gene in the DNA of a female patient (P3); this substitution is a heterozygous mutation supported by 133 out of the 243 reads that spanned this site (Figure 5D). To predict the influence of these two variants on protein function, we also performed an analysis of evolutionary conservation using the ClustalW tool. This analysis indicated that both of the novel variants were highly conserved across species. In addition, SIFT prediction demonstrated that these two mutations were in fact damaging mutations (Table 2). The current data suggest that these two variants may play important roles in gene function (Figure 5E and 5F).

### Confirmation of the identified variants by Sanger sequencing in 7 samples

**Figure 6:**
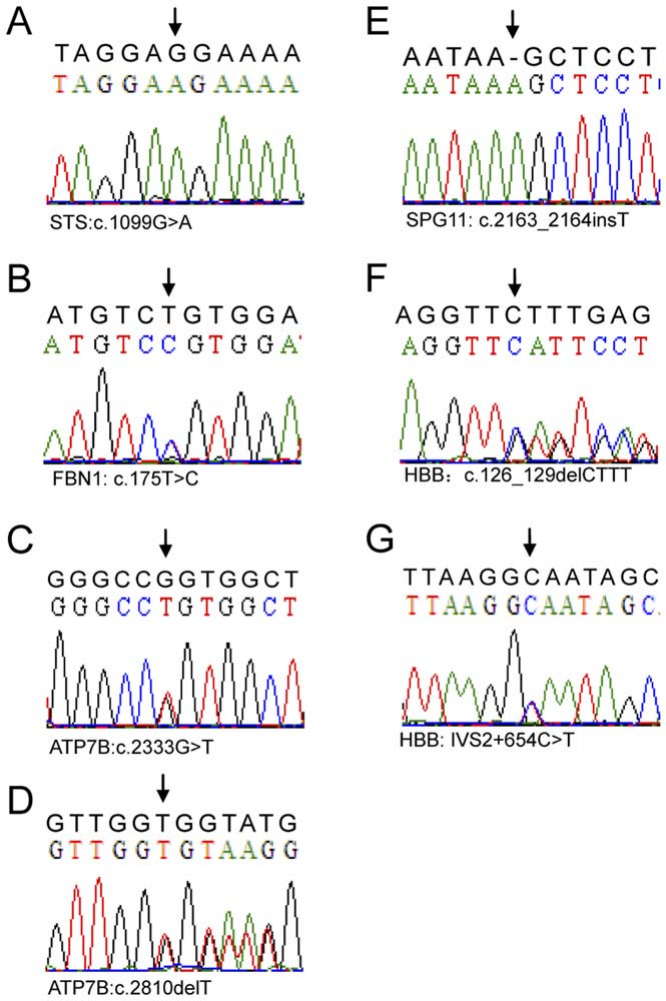
Verification of identified disease-causing mutations by the classical Sanger sequencing method. (A) A homozygous missense substitution of G with A was confirmed in the STS gene in patient P2, who has X-linked ichthyosis. (B) Double peaks indicating a heterozygous missense substitution mutation (c.175T>C) were verified in the FBN1 gene in patient P3, who has Marfan syndrome. (C and D) A heterozygous missense substitution of G with T and a heterozygous deletion mutation were confirmed in patient P4 with Wilson's disease. (E) A homozygous insertion mutation in the coding region was verified in the patient suffering from HSP-TCC disease. (F) A short, heterozygous deletion of 4 base pairs was verified in patient P5, who has β-thalassemia. (G) The heterozygous substitution of C with T at the intron 2 splice site was validated by Sanger sequencing for patient P6, who has β-thalassemia. The black-colored sequences indicate the reference sequences.

Using the Targeted DNA-HiSeq method described in this study, we identified 7 potential mutations in 6 disease-causing genes, including five previously reported missense or small insertion or deletion variants in 4 patients (P1, P4, P5 and P6), two novel missense mutations in P2 and P3 and one exon deletion mutation in P7 (Table 2). The known homozygous SPG11 gene mutation (c.2163_2164insT, MIM# 610844) was detected in patient P1 and was supported by 234 out of the 235 reads [20]. Patient P4, with the rare inherited disorder Wilson's disease, presented two known heterozygous ATP7B mutations (c.2333G>T, p.R778L; c.2810detT, MIM# 606882); 127 of the 254 reads at this locus carried the prevalent substitution mutation, and almost half of the reads included the deletion mutation [21], [22]. In the two patients suffering from β-thalassemia, we identified two different mutations in the β-subunit of the hemoglobin gene (HBB, MIM# 141900): 126_129delCTTT (269 variants and 421 wild types reads) in patient P5 and IVS2+654C>T (130 of 255 reads) in patient P6 [23], [24]. To confirm the accuracy of the potential mutations identified by Targeted DNA-HiSeq, Sanger sequencing based on PCR was performed to analyze the 7 potential mutations in the 6 disease-causing genes in the same specimens. The results showed complete consistency between the two methods (Figure 6A–6G), suggesting that the Targeted DNA-HiSeq method used in this study provides high accuracy and a very low false-positive rate.

## Discussion

Targeted next-generation sequencing has gradually been applied to simultaneously identify genetic variants in a number of individuals and provides a reliable measure of target regions and their potential variants [25]. In this study, we established a next-generation sequencing technology combined with enrichment of targeted genes, and this approach was used to detect variants in 3382 exons of 193 genes ultimately resulting in distinct hereditary diseases that has a higher incidence in the Chinese population [e.g., a high prevalence of carriers of α-thalassemia (8.53%), β-thalassemia (2.54%), and both α and β-thalassemia (0.26%)]. Overall, 11.07% of the population in this area was comprised of heterozygous carriers of α and β-thalassemia [26]. The current results have demonstrated that this approach can identify DNA variants with a maximum FPV of 3.2%. The average sequencing depth for exons is greater than 400×, and the maximum coverage of the targeted fragments is 99.54%. Seven patients with six different genetic diseases resulting from six disease-causing genes were selected to evaluate this technology, and 100 samples from normal human adults were used as controls. The resulting data showed that on average, 99.14% of the 3382 exons with more than 30-fold coverage were successfully detected using this technology. We found a total of six known variants in four disease-causing genes and two novel mutations in another two disease-causing genes (STS gene for XLI, FBN1 gene for MFS) as well as one exon deletion mutation in the DMD gene. All of the candidate mutations were confirmed by the MOLDI-TOF-MS method, thereby demonstrating the high accuracy of our method. However, in addition to the seven mutations identified in these samples, 72 potential mutations in 43 genes were identified in the same seven specimens by our method. Each patient carried 10 to 17 (average 13.1) potential mutations that were not in the seven disease-causing genes according to the OMIM database. Among these mutations, 71 were heterozygous, and 1 was homozygous. We found that 27 potential mutations were reported to be related to 16 different kinds of human diseases, and of these mutations, 51.8% were autosomal dominant (AD), 40.7% were autosomal recessive (AR), and 7.4% were X-linked recessive (XR) (Dataset S3). These results suggest that our annotated genetic polymorphism database does not present the highest possible level of representativeness and comprehensiveness, which ultimately influences our judgment regarding the identification of the variants. In the future, we will increase the number of specimens from the control group to obtain a larger polymorphism dataset. Our results also suggest that one so-called monogenic gene disease may be associated with multiple disease-causing genes rather than only one disease-causing gene.

Enrichment of targeted genes in combination with high-throughput sequencing has been used in the discovery of theoretical human disease-causing variants, though this approach has some deficiencies. The genomes of endothermic vertebrates are characterized by an abundance of CpG islands, the frequency of which increases in genomic regions with a higher GC content [27]. Highly GC-rich intervals are difficult to capture and/or sequence, as has been previously reported [28]. We calculated the effect of the target sequence GC content on sequencing depth and found a positive correlation (p<0.001, chi-squared test). More than 36% of exons with a high GC content (>75%) or low GC content (<25%) presented a sequencing depth less than 30 fold (Figure S2A and S2B). When the GC content was greater than 80%, only 14% of the exons could be successfully captured and/or sequenced to a median depth of greater than 30 fold by our method. Among the 3382 exons, 29 exons were sequenced, with a median depth of less than 30-fold across the ten samples (Table S4). In this set, 19 exons were found in regions with a high or low GC content, while only 10 exons were found in regions with a normal GC content. There was a significant difference between the two groups (p<0.001, z-test). However, this phenomenon may be related to PCR amplification efficiency. As previously reported, PCR amplification efficiency is lower in regions with a high GC content [29]. Because PCR was employed in library construction, this approach can affect the pre- or post-capture and sequencing processes in our Targeted DNA-HiSeq system. Based on these caveats, we determined that a failed read of genes with a high GC content was closely associated with PCR amplification efficiency in both probe capture and sequencing processes. To address this issue, we suggest increasing the denaturation temperature to 98°C to successfully amplify DNA fragments despite having a GC content exceeding 78% [30].

Hybridization to a solid-phase array takes almost three days (65–72 hours) and is a time-consuming process, making array-based hybridization technology slightly slower for clinical diagnoses compared with solution-based approaches, which require approximately 24 hours. Additionally, array-based methods are hampered with regard to further optimization due to their technological complexity. If a probe has to be added or changed, the whole array must be synthesized *de novo*. A combination of array- and solution-based platforms for different clinical purposes needs to be further developed and will provide a favorable alternative for certain clinical purposes. Multi-sample pooling is one way to reduce the cost of large-scale genotyping-based disease association research [31], and the efficiency and cost-effectiveness of the use of this method in next-generation sequencing has been evaluated [32]. The 2.1 Mb Nimblegen array used in the method developed in this study has a target capture size of up to 50 Mb, and the total amount of data produced for the 193 genes of one individual is ∼1.54 Mb, suggesting that a maximum of 23 individuals can be pooled together on one chip with a capture efficiency of ∼72%. Pooled sample sequencing can be used to detect both novel sequence variants and rare mutations [33].

In summary, targeted DNA-HiSeq combines next-generation sequencing with the capture of sequences from a relevant subset of “high interest” genes. This method was tested by capturing sequences from a DNA library through hybridization to oligonucleotide probes specific for 193 genetic disorder-related genes and was found to show high selectivity, to improve mutation detection enabling the discovery of novel variants, and to provide more indel data. Thus, targeted DNA-HiSeq can be used to analyze the gene variant profiles of monogenic diseases with high sensitivity, high fidelity, high throughput and high speed.

## Supporting Information

Figure S1 Estimation of the accuracy of Targeted DNA-HiSeq according to the following criteria: (1) sites are supported by at least 10 reads; (2) variant sequences are supported by more than 14% of the total reads for the heterozygous genotype; and (3) a quality value of more than 20 per base was obtained. (A) Venn diagrams of variant numbers in C1 indicated by Targeted DNA-HiSeq and in dbYH. Based on the reference genome, we found 1301 SNVs of 193 genes in YH DNA using Targeted DNA-HiSeq, of which 95.70% (1245/1301) were also identified in dbYH. (B) The distribution of ratio of candidate variants with heterozygous genotypes between common variants (1245 variants) and specific variants (56 variants) showed that the ratio of candidate variants with heterozygous genotypes was greater than 20% in 99.6% (1240/1245) of common variants.
(TIF)

Figure S2 Influence of GC content on sequencing. (A) Distribution of the median sequence coverage for exons across 10 samples corresponding to the GC content. MSCs, corresponding to the GC content, showed a normal distribution-like behavior and decreased whenever the GC content of the exon was too high or low. (B) Histogram of the proportion of exons with <30× MSC when compared with the number of exons in each GC% range. In exons with <25% or >75% GC content, more than 35% of the exons showed <30× MSC (n = 3382, p<0.001, chi-squared test). MSCs: median sequence coverage across 10 samples.
(TIF)

Table S1 The list of 193 genes causing distinct hereditary diseases with a higher incidence in the Chinese population.
(XLS)

Table S2 The information of DNA probes designed for the 193 genes including 3382 exons plus their flanking sequences.
(XLS)

Table S3 The list of our own polymorphism database for these 193 genes in 100 blood samples from 100 human adults.
(XLSX)

Table S4 The list of 29 exons with a median depth of less than 30-fold across the ten samples.
(XLS)
