# Supplementary figures and images for: Identification of Sequence Variants in Genetic Disease-Causing Genes Using Targeted Next-Generation Sequencing
Source: PLoS One. 2011 Dec 21;6(12):e29500. doi: 10.1371/journal.pone.0029500 (PMC3244462; doi:10.1371/journal.pone.0029500)

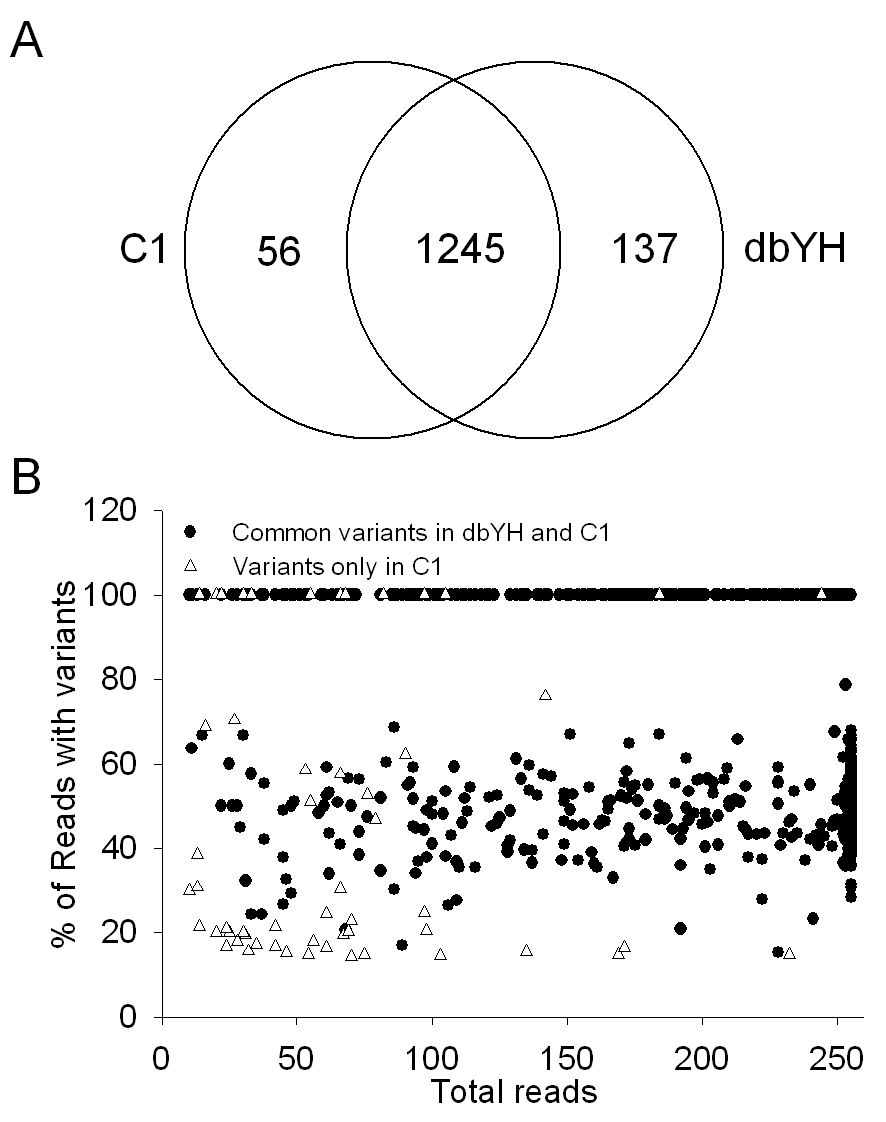

Supplement: Figure S1 — Estimation of the accuracy of Targeted DNA-HiSeq according to the following criteria: (1) sites are supported by at least 10 reads; (2) variant sequences are supported by more than 14% of the total reads for the heterozygous genotype; and (3) a quality value of more than 20 per base was obtained. (A) Venn diagrams of variant numbers in C1 indicated by Targeted DNA-HiSeq and in dbYH. Based on the reference genome, we found 1301 SNVs of 193 genes in YH DNA using Targeted DNA-HiSeq, of which 95.70% (1245/1301) were also identified in dbYH. (B) The distribution of ratio of candidate variants with heterozygous genotypes between common variants (1245 variants) and specific variants (56 variants) showed that the ratio of candidate variants with heterozygous genotypes was greater than 20% in 99.6% (1240/1245) of common variants. (TIF) [file pone.0029500.s001.tif]

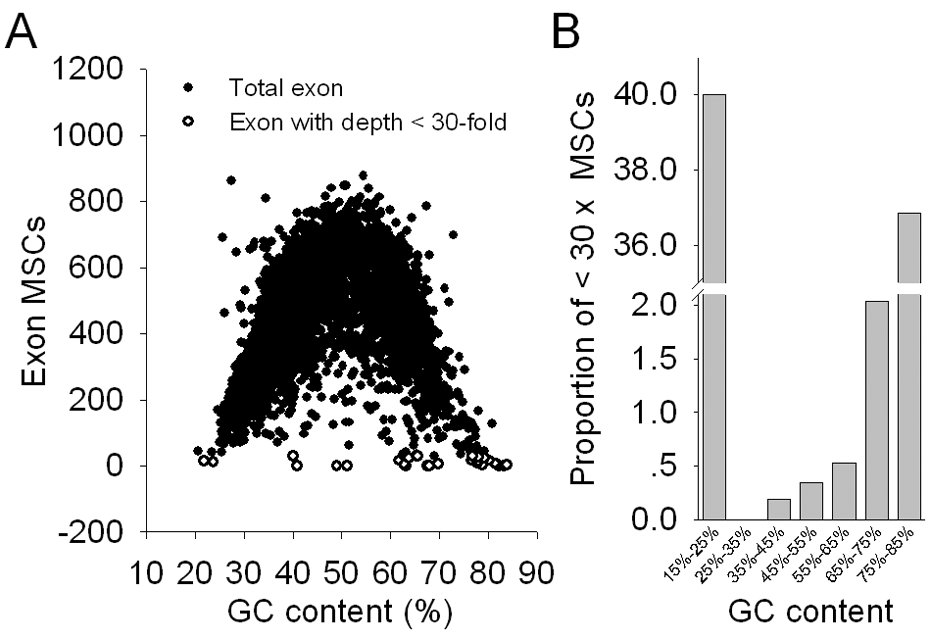

Supplement: Figure S2 — Influence of GC content on sequencing. (A) Distribution of the median sequence coverage for exons across 10 samples corresponding to the GC content. MSCs, corresponding to the GC content, showed a normal distribution-like behavior and decreased whenever the GC content of the exon was too high or low. (B) Histogram of the proportion of exons with <30× MSC when compared with the number of exons in each GC% range. In exons with <25% or >75% GC content, more than 35% of the exons showed <30× MSC (n = 3382, p<0.001, chi-squared test). MSCs: median sequence coverage across 10 samples. (TIF) [file pone.0029500.s002.tif]
